# Enhancement of immune surveillance in breast cancer by targeting hypoxic tumor endothelium: Can it be an immunological switch point?

**DOI:** 10.3389/fonc.2023.1063051

**Published:** 2023-03-28

**Authors:** Juvin Ann Thomas, Athira Gireesh Gireesh Moly, Hima Xavier, Priya Suboj, Amit Ladha, Gaurav Gupta, Santosh Kumar Singh, Partha Palit, Suboj Babykutty

**Affiliations:** ^1^ Centre for Tumor Immunology and Microenvironment, Department of Zoology, Mar Ivanios College, Nalanchira, Thiruvananthapuram, Kerala, India; ^2^ Department of Botany and Biotechnology, St. Xaviers College, Thumba, Thiruvananthapuram, Kerala, India; ^3^ School of Biosciences, University of Birmingham, Edgbaston, Birmingham, West-Midlands, United Kingdom; ^4^ Department of Immunology, University of Manitoba, Winnipeg, MB, Canada; ^5^ Centre of Experimental Medicine and Surgery, Institute of Medical Sciences, Banaras Hindu University, Varanasi, India; ^6^ Drug Discovery Research Laboratory, Assam University, Silchar, Department of Pharmaceutical Sciences, Assam, India

**Keywords:** tumor endothelial cells (TECs), hypoxia inducible factors (HIFs), myeloid derived suppressor cells (MDSCs), T regulatory cells (Treg cells), angiogenesis, hypoxic tumor microenvironment, immunological switch point

## Abstract

Breast cancer ranks second among the causes of cancer-related deaths in women. In spite of the recent advances achieved in the diagnosis and treatment of breast cancer, further study is required to overcome the risk of cancer resistance to treatment and thereby improve the prognosis of individuals with advanced-stage breast cancer. The existence of a hypoxic microenvironment is a well-known event in the development of mutagenesis and rapid proliferation of cancer cells. Tumor cells, purposefully cause local hypoxia in order to induce angiogenesis and growth factors that promote tumor growth and metastatic characteristics, while healthy tissue surrounding the tumor suffers damage or mutate. It has been found that these settings with low oxygen levels cause immunosuppression and a lack of immune surveillance by reducing the activation and recruitment of tumor infiltrating leukocytes (TILs). The immune system is further suppressed by hypoxic tumor endothelium through a variety of ways, which creates an immunosuppressive milieu in the tumor microenvironment. Non responsiveness of tumor endothelium to inflammatory signals or endothelial anergy exclude effector T cells from the tumor milieu. Expression of endothelial specific antigens and immunoinhibitory molecules like Programmed death ligand 1,2 (PDL–1, 2) and T cell immunoglobulin and mucin-domain containing-3 (TIM-3) by tumor endothelium adds fuel to the fire by inhibiting T lymphocytes while promoting regulatory T cells. The hypoxic microenvironment in turn recruits Myeloid Derived Suppressor Cells (MDSCs), Tumor Associated Macrophages (TAMs) and T regulatory cells (Treg). The structure and function of newly generated blood vessels within tumors, on the other hand, are aberrant, lacking the specific organization of normal tissue vasculature. Vascular normalisation may work for a variety of tumour types and show to be an advantageous complement to immunotherapy for improving tumour access. By enhancing immune response in the hypoxic tumor microenvironment, *via* immune-herbal therapeutic and immune-nutraceuticals based approaches that leverage immunological evasion of tumor, will be briefly reviewed in this article. Whether these tactics may be the game changer for emerging immunological switch point to attenuate the breast cancer growth and prevent metastatic cell division, is the key concern of the current study.

## 1 Introduction

The most prevalent types of cancer diagnosed and the main reason for deaths due to cancer among women is breast cancer (BC) (1). The detection and treatment of BC have undergone a number of advancements in recent years. Since the early 1990s, there has been a 39% decrease in breast cancer mortality thanks to a combination of better screening, earlier detection/diagnosis, and anti-cancer medicines that have made substantial advances. The prognosis for patients with advanced-stage breast cancer can be improved, though further investigations are required to overcome the threat of cancer resistance to treatment.

Aggressive breast cancers with hypoxic cores account for 40% of cases; these tumours are highly metastatic, and resistant to the majority of treatments. Breast cancer stem cells (BCSCs) multiply in a hypoxic tumor microenvironment, which results in a variety of epigenetic changes that retain cancer stem cells in an undifferentiated state and aid in its development and recurrence (2).Unchecked tumor cell growth outgrows the surrounding vascular system, which leads to a reduction in oxygen delivery relative to demand. Chronic hypoxia refers to this restriction on oxygen diffusion, whereas acute hypoxia occurs when blood arteries abnormally close, leading to reduced perfusion (3–5). Chronic hypoxia causes DNA breakage, malfunctions in the mending systems and mutagenesis (6). On the other hand, short-term hypoxia boosts the generation of Reactive Oxygen Species (ROS), tumor survival, and spontaneous metastasis (7, 8). Cancer cells have demonstrated radio-resistance *in vitro* and *in vivo* under both chronic and acute hypoxia (9). Hypoxia-inducible factors (HIF), which is a transcription factor, serves as the catalyst for tumor growth (10, 11). HIFs are heterodimers made up of the oxygen-sensitive HIF–α and a subsequently expressed HIF–β (12). There are three isoforms; HIF1 and HIF2 are well known, while HIF 3 is also present but has not been well researched in relation to cancer (13). HIF-1 and HIF-2 under normoxic circumstances undergo hydroxylation at particular proline residues. This causes the tumor suppressor protein Von-Hippel Lindau (VHL) protein to bind to HIF 1 and 2, which then makes it easier for them to be degraded by the ubiquitin-proteaosome system (11, 14, 15). A variety of genes encoding proteins are used for anaerobic energy production, vascularization, extracellular matrix (ECM) remodelling, suppression of apoptosis, and metastasis progression. They are transcribed when hypoxia occurs due to which HIF–1 and HIF–2 get dimerized with the subunit. They (HIF–1 and HIF–2) are then translocated to the nucleus and where they activate hypoxia responsive elements (HRE) (11, 16). Patients with breast cancer who had higher levels of HIF expression in their primary tumor biopsies are more likely to develop metastases to the bone, lungs, liver, brain, and local lymph and causes major portion of breast cancer related mortalities (17).

Through a series of sequential multistep processes the original tumour transforms into a secondary tumour at a distant site (18). The EMT transcription factors (EMT-TFs), mainly belonging to the SNAIL, TWIST, and ZEB families, which play significant roles in all processes of tumour metastasis, cause the epithelial to mesenchymal transition (EMT), which is critical for cancer spread (19).

EMT type-1, type-2, and type-3 subtypes have been extensively investigated in a variety of physiological and pathological processes. Type -1 EMT is the exchange of Epithelial to Mesenchymal cells and associated with events in embryonic phase such as implantation, embryogenesis and organ development, while Type -2 is the transformation of epithelial to mesenchymal cells that occurs during wound healing and fibrosis driven by inflammation. Type-3 EMT is the transition of epithelial cells to mesenchymal cells and is active in different types of cancers including breast cancer. Therefore, Type-3 EMT is also known as “oncogenic epithelial-mesenchymal transition” (20). Different subtypes of breast cancer show distinct metastatic organ tropisms governed by different molecular mechanisms. Along with distant lymph nodes, common target organs for breast cancer metastasis include the bone, lung, liver, and brain (21). Though all breast cancer subtypes show bone metastasis, Luminal A and B have bone as their major metastatic site. Luminal B subtype is more probable than luminal A subtype to have bone as the first site of relapse when compared to other subtypes. Incidence of bone metastasis is much higher in luminal subtype tumors than in HER-2 positive and basal like subtypes. HER -2 positive subtype is more often positively tropic to liver and luminal B and basal-like subtypes present higher levels of lung -specific metastasis (22). Triple negative breast cancer (TNBC) is often associated with visceral metastases including lung, liver and brain (23). Additionally, it has been reported that hypoxia triggers EMT in different type of cancers including breast cancer, prostate cancer and oral cancer (24). Studies by Peng, Jianheng, et al. (2018). clearly show that TGF-1 and Suppressor of Mothers Against Decapentaplegic (SMAD3) expression levels were both dramatically raised by HIF-1 in breast cancer cells, however SMAD3 overexpression had no effect on either of these proteins’ expression levels (25). Moreover, HIF-1α upregulated the expression of EMT transcription factors, SWIFT and SNAIL. In case of SWIFT in breast cancer cell lines, HIF-1α could directly bind to proximal promoter of SWIFT and enhance transcription (26). Hypoxia triggers a significant up-regulation of angiogenic growth factors and their receptors, which causes endothelial cell migration with enhanced vascular permeability and promotes tumor angiogenesis. Due to the leaky vessels and haphazard arrangement, tumor and stromal cells have limited access to nutrients and oxygen during transformation and proliferation (27). However, tumors make up for this by producing metabolic intermediates that act as precursors for biosynthetic pathways, which allows cancer cells to adapt to these circumstances in the tumor microenvironment (TME) and continue to grow and multiply. Glucose metabolism is switched from the tricarboxylic acid pathway to the oxygen independent glycolysis through the activation of glycolytic pathway regulators such as glucose transport proteins (GLUTs), hexokinase 1 and 2 (HK1, 2), and pyruvate dehydrogenase kinase 1 (PDK1) (28–30). The role of HIF–1 in reshaping the topography of the ECM under hypoxia by collagen deposition and promoting the increased expression of remodelling enzymes such prolyl-4-hydroxylases and lysyl oxidases, which leads to ECM stiffness and metastasis, has been determined by studies. An increasing body of research indicates that hypoxia promotes the chemo-invasive and metastatic potential of breast cancer by activating metalloprotease 2 and 9 (MMP-2 and MMP-9), which also degrade ECM (31–33). According to multiple studies, hypoxic stress is expected to stimulate VEGF (Vascular Endothelial Growth Factor), a crucial regulator of angiogenesis (34). Immunosurveillance in breast cancer, as in many other tumor forms, is functionally represented by the presence of tumor infiltrating lymphocytes (TILs) into the neoplastic cellular mass (35). According to Ono et al., 2012, when compared to the HER–2-/HR+ subtype, TILs were significantly greater in TNBC (triple negative breast cancer) and HER–2+/HR- breast cancer subtypes. Furthermore, compared to TNBC with low TILs levels, the pathological complete response (pCR) rate was considerably higher in TNBC with high TILs scores (36). It has been found that low oxygen levels in the TME cause TILs to become less activated, which results in immunological suppression and less immune detection. Since hypoxia-associated transcription factors have long been suggested as a viable target for immunostimulatory therapy and immunological detection, numerous researchers have found therapeutic strategies for inhibiting these factors (37). In the TME, it has been demonstrated that overexpression of HIF-1 specifically affects many aspects of the immune cell’s capacity to fight tumors (38). The foregoing discussion has briefly outlined the role of hypoxia in the aggressiveness of breast cancer, specifically in the transformation of normal endothelium into tumorigenic endothelium, as well as the various therapeutic approaches by which immune modulators can specifically target breast cancer and how hypoxic tumor endothelial cells can be targeted within the TME.

## 2 Hypoxia- induced immunosuppression in tumor microenvironment

**Figure 1:**
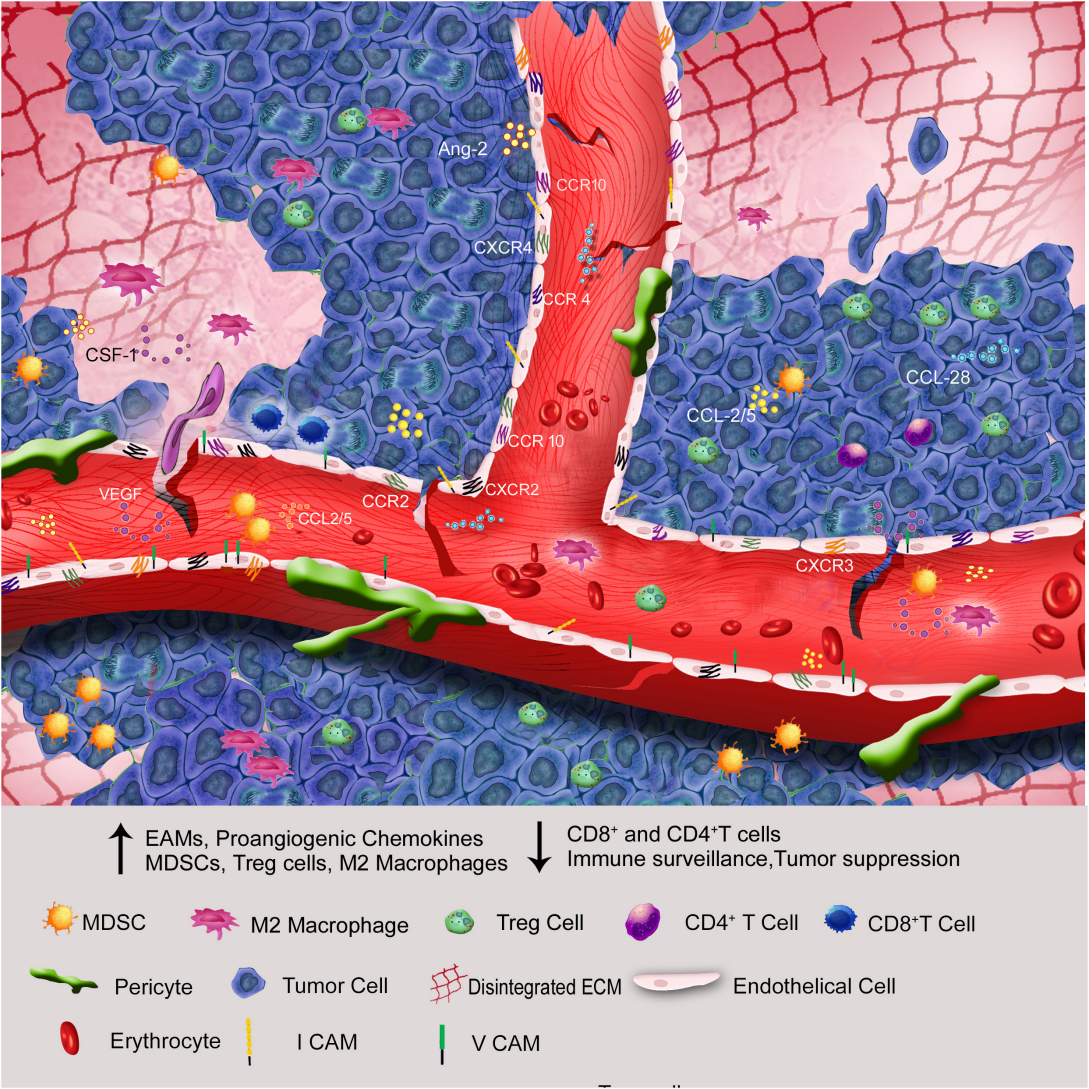
Through endothelial anergy, elevation of proangiogenic chemokines, cell adhesion molecules, and ECM disintegration, the hypoxic core of solid tumors attracts more immunosuppressive cells than immunostimulatory cells to the tumor microenvironment.

Growing body of data indicates that, a hypoxic microenvironment may protect tumors from immunological treatments as well as naturally occurring anti-tumor immune responses by limiting anti-tumor immune effector cells and encouraging immune escape. It is clear that hypoxia affects immune cells either directly or indirectly, supporting the TME in a direction that is immunosuppressive (39–41). Understanding the disease biology of Breast Cancer (BC) requires an understanding of the immune surveillance in the tumor microenvironment since it has the potential to either facilitate the eradication of disease or encourage tumor growth. This dual role is sustained by the dynamic interactions between diverse immune effector cells, tumour cells, stromal cells, and soluble substances in the tumour microenvironment (42). Selection favours tumor variants with the genetic trait of escaping immunological detection, while variants lacking it are wiped off by immune surveillance. In addition to genetic variation, deficiencies in antigen presentation methods, T-cell receptor (TCR) signalling, interferon (IFN) signalling pathways, and expression of the MHC class I protein has been lost or mutated, along with tumour antigens (43, 44), which will impair the immune surveillance mechanism in the tumor microenvironment. Inhibition of tumor antigen-specific T cells by intratumoral myeloid-derived suppressor cells (MDSCs), T regulatory cells (Tregs), and the switch from an anti-tumorigenic T-helper type 1 (TH1) to a pro-tumorigenic T-helper type 2 (TH2) immune response are additional factors that significantly support tumor growth. Furthermore, a variety of soluble factors like TGF-β, IL–10 etc are released by tumor/stromal cells inhibit T-cell activation and dendritic function while promoting stromal remodelling and angiogenesis (45, 46). The hypoxic core of the tumors attracts more pro-tumorigenic leukocytes, including MDSCs, tumor-associated macrophages (TAMs), and regulatory T cells. This double down the body’s natural immune defensive measure to evacuate the aggressive tumor (47) **(** **Figure 1** **)**. An in-depth study using Balb/c mice found that intra-tumoral hypoxia increases HIF-1 activation, which then sequentially activates the PDL-1, CD73, and CD47 genes to reduce the recruitment and activity of CD8+ T cells, NK cells, and macrophages, ultimately causing activity to evade both innate and adaptive immunity (48). Chemokines and their related receptors are another important hypoxic hotspot (49), which in turn affect tumor endothelial cells and increase the over expression of VEGF, CXCL12, and its receptor CXCR4, making all of them function on endothelial cells in an autocrine way. The expression of the pro-inflammatory chemokines CCL5 and CCL2 increased in HIF–1-deficient tumors, which in turn increased the infiltration of cytotoxic lymphocytes into the tumor (50). Additionally, the CXCR4-CXCR12 axis promotes metastasis to distant organs (51). Thus, hypoxic stress in TME along with activation of HIF transcription factors, is a principal cause for breast cancer angiogenesis, metastasis, immune suppression and overall poor survival rate. In the subsequent sections of this review, a detailed mechanism of hypoxia induced vascular abnormalities that activate endothelial cells (ECs) associated with tumor cells and immune suppression is furnished.

## 3 Hypoxia, a prerequisite to vascular alterations in the tumor microenvironment

Tumor vasculature is different from healthy blood vessels in a number of aspects, including irregular structural dynamics, high permeability, and convoluted arteries, whereas healthy blood vessels are well-organized and provide for the best perfusion of nutrients and oxygen (52). Specialized mural cells called pericytes are seen in the normal vasculature, whereendothelium covers them uniformly (53). Patchy hypoperfusion and blood vessel leakage are caused by the loosening, unstable, and tumor-related pericyte phenotype (54).

**Figure 2:**
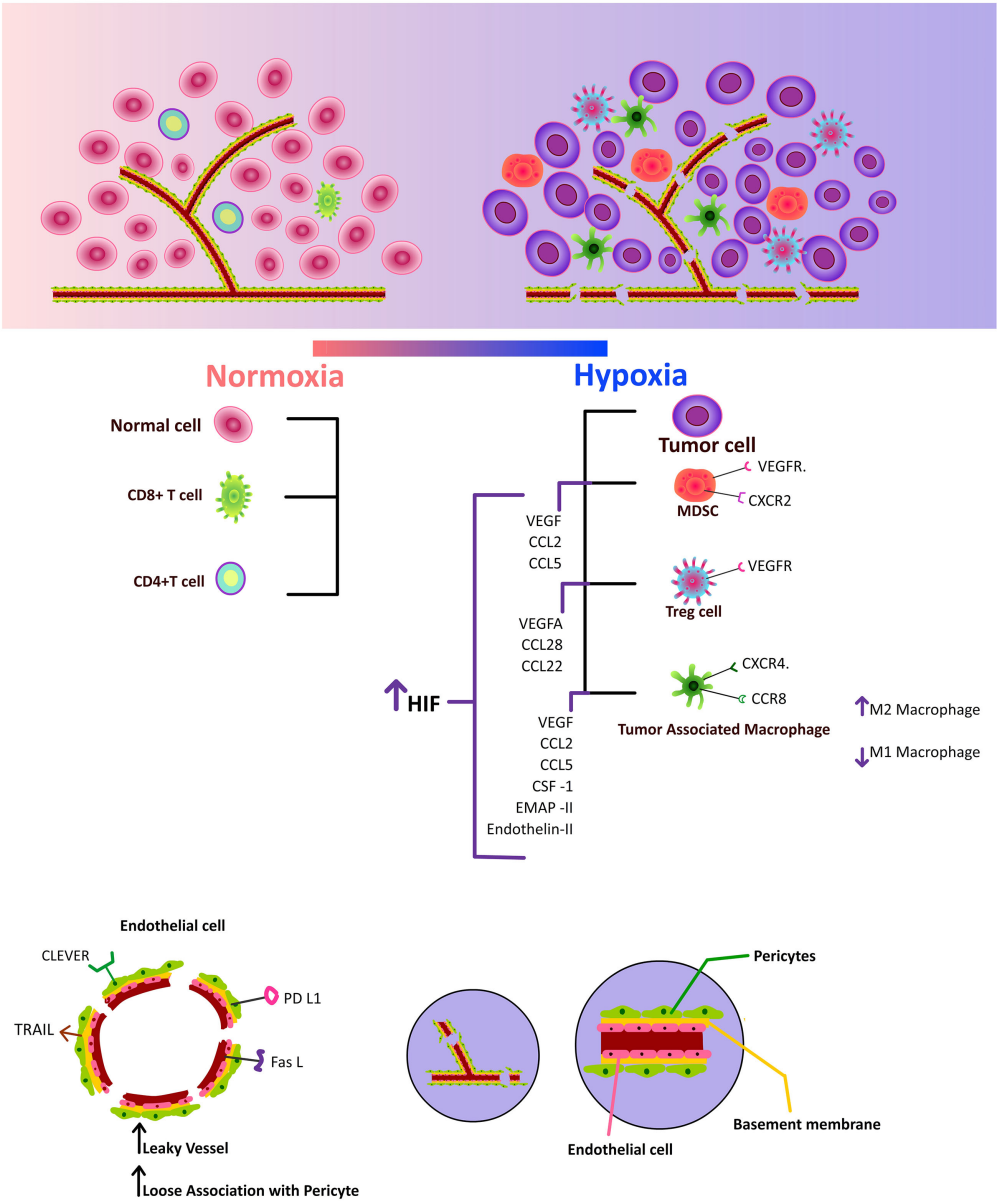
Increased hypoxia inducible factor, which upregulates pro-angiogenic factors and chemokines, draws immunosuppressive cells to the tumor microenvironment. Additionally, increased CLEVER, TRAIL, PD-L1, and FAS-L expression on tumor endothelium impairs effector T cells.

Endothelial cells interact with tumor cells in numerous ways to promote angiogenesis (55) **(** **Figure 2** **)**. They serve as the connecting link between cancer cells and immune cells (56). Proangiogenic substances like Vascular Endothelial Growth Factor (VEGF), basic fibroblast growth factor (bFGF), placental growth factor (PGF), and angiopoietin are released by tumor cells to initiate angiogenesis. According to studies, the hypoxic tumor microenvironment might boost VEGF synthesis, which in turn promotes the growth of new blood vessels (57). When the microenvironment experiences a lack of oxygen, endothelial cells and pericytes are stimulated to create VEGF, which functions in an autocrine and paracrine manner to increase the recruitment and activation of endothelial cells in the tumor site (58). Angiopoietin-2 (Ang–2) plays a critical function in destabilising vasculature for normal or pathological angiogenesis and is also up-regulated by hypoxia. It is only expressed at sites of vascular remodelling. Numerous studies have documented the essential function of Ang-2, a ligand for the endothelial cell-specific tyrosine kinase Tie2, in the vascular permeability and blood vessel instability that leads to tumor growth (59, 60). Thus, newly formed blood vessels have an uneven thickness of the basement membrane, a loose interaction between pericytes and endothelial cells, an increase in interstitial pressure, and ultimate vascular leakage (47). One of the main processes behind angiogenesis is the angiopoietin/Tie (tyrosine kinase) signalling pathway, which is composed of growth factors called angiopoietins. These include Ang-1, a strong angiogenic growth factor that communicates with Tie2, and Ang-2, a vascular disruptor with a negative effect that also uses Tie2 as a conduit (61). To cause pericyte separation from the basement membrane and migration, Ang-2 and Tie2 bind in the hypoxic tumor microenvironment. Mice lacking in pericytes had higher Ang–2 levels, suggesting that pericytes may control Ang–2 levels and limit vascular permeability. This finding highlights the importance of Ang–2 in reducing vessel leakiness (62, 63). In a different study, it was reported that reduced pericyte coverage increased IL-6 expression in the hypoxic tumor microenvironment and MDSC transmigration and circulating malignant cell phenotype (64). Rgs5 overexpression in pericytes and endothelium seen in the hypoxic tumor microenvironment causes the vasculature to become unstable. Better pericyte coverage, less vascular leakage, and adequate oxygen perfusion was all observed in the Rgs5-deficient mice (65). In-depth research found that the development of a receptor complex made up of PDGF-R and VEGF-R2 during PDGF-induced angiogenesis is what causes VEGF to activate VEGF-R2 and suppress PDGF-R signalling in vascular smooth muscle cells (66), which then decouples neo-vasculature from pericyte covering. Researchers have shown that inhibiting VEGF and Ang–2 jointly results in tumor necrosis, vascular regression, intra-tumoral phagocyte antigen presentation, and a reduction in breast cancer-brain metastases (67, 68).

## 4 Endothelial cells in tumors are the “switch point”, aren’t they?

Endothelial Cells (ECs) ECs have specific functions based on their locations and exhibit distinct heterogeneity across vascular beds. They have a critical role in the regulation of immune responses, inflammation, angiogenesis and actively control the degree of vascular relaxation and constriction (69). ECs that line tumor blood vessels are initially derived from the surrounding tissue, and in due course of the tumor progression, they reprogram to a tumoral phenotype (70). Normal physiologic conditions do not often need the activation of ECs, but in tumors, hypoxia and other inflammatory signals cause ECs in the tumor microenvironment to become active, causing aberrant angiogenesis and impeding normal immune surveillance (71). Numerous inflammatory reactions in the tumor microenvironment, such as inflammatory cytokines, chemokines, reactive oxygen species, etc., directly or indirectly stimulate the tumor endothelium. Tumor endothelium has a very different genetic profile than healthy endothelium **(** **Table 1** **)**, with the main variations influencing a number of cell adhesion molecules (such as ICAM1, VCAM1, E-selectin), antigen presentation, and chemokines (such as CCL2, CCL18, CXCL10, and CXCL11) and cytokines involved (such as TNFα, IFNγ, and IL-1) in immune cell recruitment. All of these elements have a deleterious impact on the immune surveillance of tumor cells in the tumor microenvironment. This unique character of TEC that enables them to avoid immune cell extravasation and, unresponsiveness of TECs to pro-inflammatory stimulation is Endothelial cell anergy, also called vascular immune checkpoint (72). ECs lining tumour blood arteries have a very different metabolic profile from ECs in normal tissue. A stronger dependence of tumour ECs on glucose metabolism is supported by the transcriptional elevation of the glycolytic pathway gene, PFKFB3, in comparison to other normal EC. According to a report, tumor ECs and tumor associated macrophages (TAMs) engage in fierce competition for glucose, and the tumor ECs’ intake of the metabolite boosts the angiogenic response in the tumor microenvironment (73). TECs produce energy through aerobic glycolysis and fatty acid oxidation, rather than oxidative phosphorylation. This metabolic reprogramming enables the tumor ECsto check the production of reactive oxygen species (ROS), and also allows the production of ATP more rapidly than through oxidative metabolism (74). Alam et al. (2014) has identified suprabasin as a new marker for TECs. Suprabasin, an upstream component of the AKT pathway, was substantially expressed in TECs compared to normal ECs and linked favourably with TECs’ capacity for migration and tube formation (75). On the other hand, microarray and immunohistochemical studies revealed that biglycan is a specific marker of TEC and an autocrine angiogenic factor of TECs (76). Because of the aggressive behaviour of tumor endothelial cells and their particular molecular, cytogenetic, and metabolic characteristics, the tumor microenvironment can therefore selectively draw in immune suppressive cells. The role of TECs in increasing immunological suppression is discussed in the following sections of this review.

**Table 1:** Factors converting normal endothelium to tumor endothelium.

Factors converting Normal endothelium to Tumor endothelium	
Antigen presentation	˙TECs act as Antigen Presenting Cells (APCs)
Recruitment of immune suppressive cells	˙Chemokines - CCL2, CCL18, CXCL10, and CXCL11 ˙Cytokines - TNFα, IFNγ, and IL-1
Endothelial anergy	˙Unresponsiveness to inflammatory stimulation ˙Downregulation of adhesion molecules- ICAM-1/-2, VCAM-1, E-selectin, and CD34
Higher dependence on glucose metabolism	˙Elevation of glycolytic pathway gene, PFKFB3. ˙Aerobic glycolysis and fatty acid oxidation
Expression of specific markers	˙Suprabasin ˙Biglycan
Overexpression of Immune checkpoint molecules	˙PDL-1 - Overexpression
Overexpression of molecules to prevent effector T cells	˙Overexpression of TRAIL and CLEVER

## 5 Tumor endothelium driven immunesupression and tumor progression

**Figure 3:**
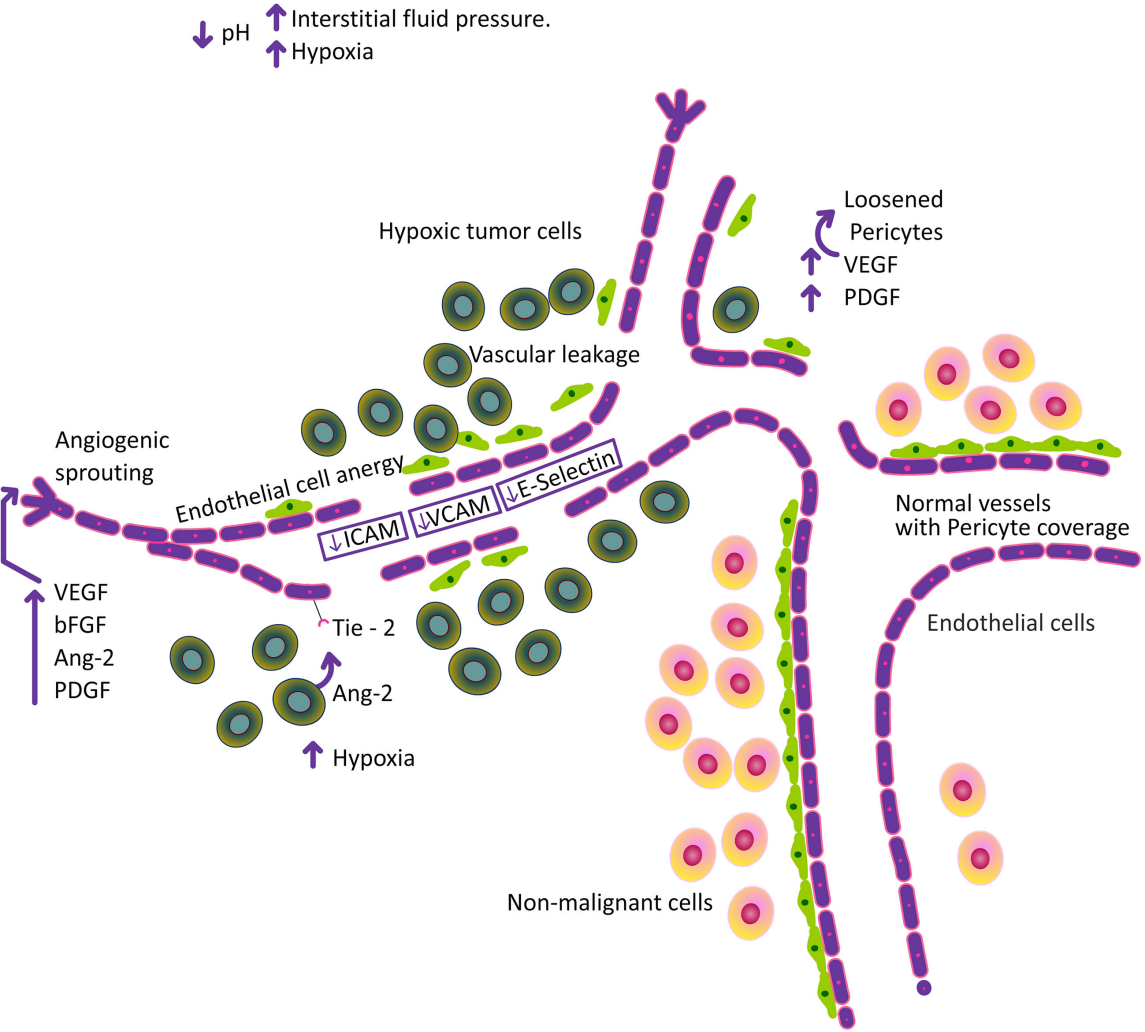
Increase in hypoxia, alter the pH and increase in interstitial fluid pressure followed by structural abnormalities in the tumor vasculature.

The success of immunotherapy depends on adequate immune cell infiltration, and low immune cell infiltration reduces the effectiveness of immunotherapy. Despite the fact that angiogenesis triggered by tumors is crucial for the growth of solid tumors, mounting evidences indicate that it also aids in immune evasion by fostering a highly immunosuppressive TME by increasing the proportion of T reg cells and MDSCs. Furthermore, by preventing dendritic cell (DC) maturation and T cell growth, VEGF impacts immunological responses in TME (77). Leukocytes are extravasated to tissues from blood arteries during typical inflammation. These leukocytes in circulation were halted and firmly adhered to the endothelium cells by a multi-step procedure. This mechanism calls for the endothelial adhesion molecules (EAM), E-selectin (rolling), ICAM1 and VCAM1 (firm rest), VE-cadherin and CD31 (trans-endothelial migration). In addition to EAMs, surface antigens (such as HLA molecules) must be upregulated, pro-thrombotic endothelial cell changes (such as the loss of the surface anticoagulant molecules thrombomodulin and heparan sulphate), cytokines (such as IL–6, IL–8, and MCP–1) production, and changes in vascular tone (such as the loss of vascular integrity and expression of vasodilators) are associated with inflammation (78). When inflammatory cytokines like TNF, IFN, and IL–1 are released, normal ECs are activated. This causes an up-regulation of adhesion molecules, which in turn triggers the extravasation of leukocytes. However, the continual secretion of pro-angiogenic molecules VEGF and bFGF in hypoxic microenvironments adversely affects this process. Even when TNF is present, they prevent ICAM1, VCAM1, and E-selectin from being overstimulated (79–81). The hypoxia inducing factors favour the heterogeneity as well as inflammation of tumor endothelial cell (82). *In vitro* and *in vivo* studies by Tellier et al. (2015) showed that ECs exposed to hypoxia expressed tumor-promoting pro-inflammatory cytokines and chemokine such as IL-6, IL-8, and CXCL1 (83). In addition, hypoxic tumor niche affects protein glycosylation and favours recruitment of immune suppressive cells to TME, thereby impacting tumor progression (84). Research shows that the tumor endothelium downregulates the expression of endothelial adhesion molecules (EAM) such as ICAM1/2, VCAM1, E-selectin, and CD34. This lessens immune cell infiltration or leucocyte extravasation in the TME, enabling the TME to adopt an immune evasion strategy and accelerate tumor growth (72, 79, 85). Hypoxic tumor microenvironment alters the tumor endothelium leading to its diversity and also induces its “stemness”. This induction of stemness is favourable for cancer stem cells, which can drive tumor initiation and progression (86). Moreover, breast cancer stem cells enhance its tumorigenic phenotype (87). Cancer stem cells and their role in tumor progression is a vast horizon and beyond the scope of this review. Stemness genes such stem cell antigen-1 (Sca-1), MDR-1, and aldehyde dehydrogenase (ALDH) have been found to have elevated expression in TECs. This tumour stromal stem-like cell population affects the TEC population in the TME (57). With higher VEGF expression, ovarian, oesophageal, and colorectal tumors showed worse survival rates and a higher chance of relapse. VEGF synthesis in tumors affects tumor behaviour by reducing T cell numbers in addition to promoting angiogenesis. In pre-clinical tumor models, it was found that using anti-VEGF antibodies increased the recruitment of T lymphocytes into the tumor microenvironment (88, 89). Increased nitric oxide (NO) in the TME has been found to upregulate VEGF in solid tumors and further regulate the expression of several adhesion molecules involved in the interaction between EC and leukocytes. FoxP3+ T regulatory (Treg) cells predominated and there was little CD8+ infiltration in solid tumors of both humans and mice that express specific Fas ligands in the tumor vasculature. Similarly, blockade of Fas-Fas L signalling increased intra-tumoral CD8+ T cells and subsequent reduction in tumor size (90). It is now understood that the tumor endothelium plays a crucial dual role in the development of the tumor by favouring a niche where inflammation is increased by upregulating EAMs and by negatively regulating the influx of leucocytes through endothelial anergy (**Figure 3**). The role of hypoxia in attracting immune suppressive cells to the TME and its relationship with cancer endothelium are discussed in the forthcoming sections of this review.

### 5.1 Myeloid-derived suppressor cells

MDSCs are one of the main immunosuppressive elements in TME. Their activation and expansion coincide with metastasis and progression in many types of cancer (91). Those patients with higher levels of MDSCs in the tumor microenvironment have exhibited higher metastatic burden and poor survival (92).Various inflammatory cytokines such as IL-13, IL-4, and transforming growth factor (TGF–β)- are linked to MDSC proliferation, whereas granulocyte-macrophage colony-stimulating factor (GM-CSF), Prostaglandin 2 (PGE2), IL-6, stem cell factor, and vascular endothelial growth factor (VEGF) are linked to MDSC activation (93).The expression of VEGF receptor on MDSCs, explains the correlation between the up-regulation of VEGF in hypoxia and the accumulation of MDSCs in the TME (94). The presence of hypoxia in the tumor microenvironment boosts the production of CCL 5, which in turn stimulates the HIF 1α and VEGF signalling pathways. Hypoxia in the tumor microenvironment increases the production of CCL 5, and which in turn activates VEGF signalling mechanism. The expression of PD L-1 in MDSCs is increased by VEGF production in the tumor microenvironment, which limits the recruitment of cytotoxic lymphocytes (85). In line with this, a related study showed that numerous MDSC chemoattractants were upregulated in the VEGF overexpression group, suggesting that the immunosuppressive effects of VEGF are partially mediated by MDSC recruitment into the tumor microenvironment (95). Similarly, lymphatic endothelial cells (LECs) have shown to recruit MDSCs to the tumor microenvironment and increase tumor progression in TNBC cells *via* pro-angiogenic receptor CXCR2 on MDSCs. According to the study, tumor-derived vascular endothelial growth factor-C (VEGF-C) stimulated LECs to produce chemokines, which in turn helped MDSCs find their way to lymph nodes. Additionally, LEC-released chemokines increased lymphatic invasion by upregulating VE-cadherin phosphorylation and junction disruption, which in turn increased serum amyloid A1 (SAA1) expression in breast cancer cells (96). In a recent study by Roberts et al. (2022) reported that co-culturing of murine TNBC-4T1 cells with MDSC and murine LECs (iLECs) in culture inserts showed an increase in MDSC invasion in the presence of iLEC (97). Monocyte/M-MDSC recruitment to malignancies is mostly facilitated by CCL2 and CCL5 (C-C motif ligand 2/5) chemokines. There is evidence that CCL2 is crucial for recruiting PMN-MDSC as well (98). Apart from endothelial cells, the absence or presence of immature pericytes, a component of vasculature also acts as a signal for MDSC recruitment in breast cancer patients (64). Taken together, MDSC play a critical role in tumor immune suppression where its activity is tightly regulated by hypoxia *via* endothelial receptors and growth factors, which overall aids in tumor progression. The trafficking of MDSCs by TECs has not been elicited clearly this far. Thus, establishing this link may offer novel targets of anti-tumor therapies.

### 5.2 Macrophages

Macrophages make up a significant portion of the leukocyte infiltrate, which is found in all cancers to variable degrees (99). Macrophages, which are derived from the blood compartment, are renowned for their flexible and variable genomes (100). Overall, macrophage matrix metalloproteinase-12 dampens inflammation and neutrophil influx in arthritis (101). Depending upon their nature of activation and the TME, macrophages can either increase or inhibit the immune responses that fight cancer. According to reports, VEGF stimulates the growth of endothelial cells in breast cancer and attracts macrophages through the VEGF receptor. The synthesis of several pro-angiogenic substances by recruited macrophages, such as VEGF, tumor necrosis factor (TNF), and thymidine phosphorylase (TP), is able to accelerate angiogenesis in the TME. This is a form of mutual activation/dependence between TAM and tumor endothelial cells in the TME (102). Tumor cells and stroma, which make up the hypoxic core of the tumor microenvironment, promote the production of VEGF, CCL2, CCL5, CSF-1, EMAP-II, endothelin-2, SEMA3A, oncostatin M, and eotaxin. The overproduction of migratory molecules promotes macrophage infiltration of the tumor (103).Macrophages can be classified as pro inflammatory-immune stimulatory (M1) or alternatively activated anti-inflammatory-immunosuppressive (M2) macrophages depending on the environment in which they are recruited.It has been shown that the recruitment of TAM into the TME is a poor prognostic indicator for overall survival and treatment effectiveness (104). In addition, non-responsive tumor EC-derived IL-6 is a cytokine that encourages macrophage M2-like polarisation in the tumor microenvironment. The EC biomarker ESM1, which is linked to a poor prognosis in human gastrointestinal and hepatocellular carcinomas, is also highly expressed in tumor ECs in a number of mouse tumor models. Furthermore, studies have shown that ESM1 induces ECs to express ICAM1, which draws and polarises M2 macrophages toward the tumor microenvironment (105–107). As tumors grows, increased levels of hypoxia cause M1-polarized macrophages to secrete less pro-inflammatory mediators including IL-1, TNFα, and CCL17 and accelerate macrophage differentiation toward the M2-like phenotype. Despite the fact that hypoxia does not affect the proportion of different macrophage subsets, it does cause the M2-like macrophage subset to activate the transcription of pro-tumor genes, including growth factors like FGF2, PDGF, and VEGF (108, 109). Therefore, the dichotomy in macrophage differentiation into M1 and M2 type can have varied outcome on tumor progression *via* hypoxia and angiogenesis machineries (110). The polarisation of macrophages from the anti-tumor M1 phenotype to the pro-tumor M2 phenotype is clearly influenced by hypoxic stress. Focusing on TAMs that are produced by hypoxia and their trafficking across tumor endothelium may be beneficial given that it has been suggested that TAMs and cancer endothelium interact. The elucidation of this mechanism might produce brand-new indicators and therapeutic outcomes that are promising.

### 5.3 T cells

Hypoxic TME can regulate T cell response in two major ways depending upon the T cell subset. Effector T cells, in the form of CD4^+^ and CD8^+^ T cells, play a critical role in resolving tumor growth by releasing inflammatory cytokines or by direct lysis of tumor cells. However, these effector T cell responses undergo immune suppression in the tumor microenvironment which is typically hypoxic (111). Multiple factors such as tumor growth factor (TGF-β), IL-10, VEGF, indoleamine 2, 3-dioxygenase (IDO) and arginase contribute to their immune suppression (112). Another mechanism by which hypoxia mediated immune suppression in effector T cells is through the loss of expression of co-stimulatory molecules (CD80, CD86 and CD40) on dendritic cells that interact with T cells and promote immune tolerance in them (113). Cytotoxic CD8^+^ T cells are capable of infiltrating tumors making them critical for tumor clearance. However, under hypoxic conditions, these CD8^+^ T cells also undergo immune suppression *via* defective antigen presentation of tumor cells through low MHC expression, down regulation of transporter associated with antigen processing protein (TAP) and tumor antigen (114–116). This tumor hypoxia specifically promotes the immunosuppressive function of T regulatory cells including its migration and activation at the tumor site (117). Treg cells are characterized by the expression of specific cell surface molecules (such as CD25, GITR, CTLA4) and nuclear transcription factor (FOXP3) (118), mediate immune suppression by downregulating activated T cell function through increased production of immunosuppressive cytokines such as IL-10 and TGF-β or *via* interaction between CTLA-4 on Tregs and CD80/86 on antigen presenting cells or by sequestering IL-2 from naïve T-cells by its IL-2 receptor (CD25) on Tregs (119). Hypoxia induced HIF-1α results in increased expression of FOXP3 in Treg cells (120) and at the same time, also promotes CCL28 in the tumor microenvironment. This CCL28 binds to its cognate receptor CCR10 on Treg cells and thereby promotes the migration of Treg cells (117). HIF-2α is also involved in Treg stability as HIF-2α-deficient Tregs are functionally defective at suppressing effector T-cell function (121). Tumor infiltration by T lymphocytes has shown to increase overall survival in different types of cancer such as colorectal, ovarian, breast and melanoma. Tumor endothelial cells act as a major barrier for the extravasation of effector T lymphocytes into the tumor niche through the downregulation of ICAM1 and VCAM1. Furthermore, TECs can increase the expression of molecules such as common lymphatic and vasculature endothelial receptor 1 (CLEVER1) on their surface to recruit immunosuppressive Treg cells (80). **(** **Figure 2** **)** Interestingly, the expression of TRAIL and FasL on TECs selectively kill effector T cells while not hampering Treg cells. The pre-clinical studies by actively immunizing tumor endothelial expressing antigens *via* DNA vaccines and protein pulsed DCs successfully inhibited tumor growth and increased the infiltration of CD8^+^ T cells in the TME. Also, DNA vaccines targeting tumor endothelial marker 1 (TEM1), specific TEC expressing antigen could escalate intratumoral infiltration of endogenous CD3+ T cells (122). Thus, tumor induced hypoxia and tumor associated endothelial cells mediate immune suppression by directly or indirectly regulating T cell functions, which promotes tumor growth and invasiveness.

### 5.4 Tregs

Immunosuppressive T cells known as regulatory T (T reg) cells control homeostasis and self-tolerance by limiting erroneous immune responses (123). One of the essential immune cells favouring tumor growth and regulating the immunesurveillance is the CD4+CD25+Foxp3+ Treg cell (124). FoxP3, a sign of Treg activity, has been proposed as a marker of tumor progression and metastasis in breast carcinoma. In order to determine the progression and prognosis of BC, measuring Tregs recruitment and activity has been proven to be a useful approach (125). Hypoxia has been proven to associate with the infiltration of regulatory T cells in breast tumor microenvironment by the upregulation of CXCR4 receptor on Tregs. Hypoxic stress induced expression of CXCR4 by activating HIF pathway has been reported in different stromal cells including endothelial cells in TME. By increasing the expression of FOXP3, a lineage transcriptional regulator of Tregs, HIF-1 may also indirectly stimulate CXCR4 expression.

By increasing the expression of FOXP3, a lineage transcriptional regulator of Tregs, HIF-1 may also indirectly stimulate CXCR4 expression. Thus, there are opportunities for clinically targeting Tregs by blocking CXCR4 to stratify patients for anti-HIF therapies (126). Mounting evidences suggests the prime role of hypoxia in stimulating the secretion of cytokines and chemo- attractants from cancer cells and tumor associated macrophages, including CCL28, CCL22 and IL-10, that recruit Treg cells from the circulation. Hypoxia induces the expression of CD 73 on various cell types including T regs, and actively involves in the generation of immunosuppressive metabolite adenosine which negatively affects T cell function (127, 128). Treg infiltration in tumor locations can be correlated with increased microvessel density and upregulation of angiogenesis indicators like VEGF in breast and endometrial malignancies, illustrating the relation between Tregs and tumor angiogenesis (127). According to Andrea Facciabene et al., hypoxic intraperitoneal tumors recruit Treg cells, which impair effector T cell activity and promote tumor angiogenesis *via* VEGF-A. Furthermore, CCL28 secreted by hypoxic tumor cells attract Treg cells in to the tumor niche. Treg cells can directly contribute to the overproduction of VEGF-A and can promote the proliferation and recruitment of endothelial cells (129). In fact, Tregs contribute to angiogenesis indirectly by inhibiting Th1 effector T cells and secreting interferon-induced chemokines like CXCL9, 10 and 11 as well as angiostatic cytokines like TNF and IFN. Another work by Andrea Facciabene et al. shown that depletion of Treg cells reduce VEGF upregulation and angiogenesis (52). Increased expression of CCR8 was noticed in tumor infiltrating Tregs compared to circulating T regs. A promising immunotherapeutic strategy for the treatment of breast cancer would involve targeting CCR8 to prevent the migration of tumor-resident Tregs (130). Targeting T reg cells in tumors using selective immunotoxin against CD 25 (Treg marker) increased CD8+ T cell-dependent antitumor immune response in experimental tumor models (131). T reg cell elimination and subsequent anti-VEGF therapy, restored IFN- production in CD8+ T cells and enhanced the antitumor response from anti-VEGF therapy in tumors (132). Although there are lot of studies depicting hypoxia mediated T reg infiltration, there are no confirmatory studies on the role of hypoxic cancer endothelium, especially breast cancer endothelium in recruiting regulatory T cells to the TME.

### 5.5 Immune checkpoint inhibitors

Recently, immune checkpoint blockade therapy has gained attention since it allows patients’ natural immune systems to combat cancer. Immune checkpoint molecules like CTLA4, PD1, PDL–1, LAG3, and TIM–3 inhibit the immune response in different tumor types at different phases of tumor development (133). A significant development in the field of cancer immunotherapy is the discovery ofproteins like programmed cell death protein 1 (PD1), programmed cell death ligand 1 (PDL–1), and cytotoxic T lymphocyte-associated antigen 4 (CTLA4). These molecules block the signals that result from the activation of the T cell receptor (TCR), which eliminates cytotoxic T cells (CTLs) and blocks anti-tumor immunity.

The FDA recently approved the use of two mouse antibodies (immune checkpoint inhibitors) known as anti-CTLA-4 and anti-PD-1 for the treatment of humans (134). Immune checkpoint (IC) molecules are found in TME cells including cancer cells, immune cells, and stromal cells like TEC. It has been discovered that TECs express PD-L1 and PD-L2 along with other well-known immunoinhibitory molecules such TIM-3, which raises the possibility that they may be able to directly suppress T cell activation when the vasculature is present. It has been shown that pro-inflammatory cytokines like IFN and TNF encourage PDL–1 up-regulation on ECs. Although the most often used biomarker in immune-oncology to determine treatment options and patient stratification is now PDL–1 expression by cancer cells, its therapeutic value has not yet been determined (82). Studies conducted *in vitro* and *in vivo* by Barsoum I et al. gave the information for the upregulation of PDL-1 in an HIF dependent way. By utilising glyceryl trinitrate (GTN), an agonist of nitric oxide (NO) for signalling, they were also able to prevent the HIF-1 accumulation and the hypoxia-dependent PDL-1 production and Cytotoxic T Lymphocyte resistance (135). The expression of PD-L1/2 may begin to make T lymphocytes lethargic and exhausted before they even enter the cancer microenvironment. In the pancreatic neuroendocrine tumour model and the polyoma middle T oncoprotein (PyMT) breast cancer model (RT2-PNET), the concomitant administration of anti-VEGFR2 and PDL–1 antibodies promoted the development of specialised vessels known as High Endothelial Venules (HEVs), which support lymphocyte trafficking and enhance T-cell infiltration. Even before T cells penetrate the tumour microenvironment, the expression of PDL–1/2 may start to cause them to become anergic and worn out. In the pancreatic neuroendocrine tumour model and the polyoma middle T oncoprotein (PyMT) breast cancer model (RT2-PNET), the combination of both anti-VEGFR2 antibodies and PDL–1 antibodies stimulated the formation of specialised vessels called High Endothelial Venules (HEVs), which contribute to lymphocyte trafficking and improved T-cell infiltration (129).

It has proven that Immune checkpoint inhibitors or drugs could increase immune surveillance. Thus, combination therapies targeting Immune checkpoint and metabolism of cancer endothelium could be a promising strategy to reduce tumor progression.

## 6 Trans-endothelial migration

Due to metastasis and disease recurrence, breast cancer is the cancer that claims the lives of more women than any other (136). Journey of tumor cells across the endothelial membrane is a key step in the process of tumor invasion and metastasis. Tumor cells cross the vascular membrane stimulated by several cytokines and growth factors such as Transforming Growth Factor-Beta (TGF-β) superfamily of proteins,Bone Morphogenetic Proteins (BMPs). This is called Endothelial-mesenchymal transition (EndMT). EndMT induced by TGF-β shows a decrease in the expression of endothelial markers such as VE-cadherin, claudin and zona-occludens 1 (ZO-1) and an elevation in EMT transcription factors such as Snail, Slug and ZEB-1 which marks metastasis. TGF-β promotes ECs to change into CAF-like cells, which results in the loss of endothelial adhesion molecules and remodelling of the endothelium cytoskeleton *via* the Rho and Rac-1 signalling pathway, which is the primary characteristic of EndMT (76, 137, 138). It has been found that hypoxia associated with inflammation or tissue damage can also cause EndEMT (139). Rokana, et al. (2021) have recently studied the role of ICAM in trans-endothelial migration in breast cancer and also assessed the therapeutic efficacy of anti-ICAM1 neutralizing antibody on breast tumor cell aggregation and trans-endothelial migration. This anti-ICAM treatment inhibited the cluster formation of TNBC cells in suspension. Clinical data also showed high levels of ICAM1 mRNA expression in breast tumors which might mediate distant metastasis. This makes ICAM as a potential therapeutic target in TNBC metastasis (140). S100P and Ezrin, two members of the S100 family of short calcium binding proteins, also encourage the trans-endothelial migration of triple negative breast cancer cells. Furthermore, S100P activity has been linked to a variety of malignancies, a poor prognosis, metastasis and recurrence, and a low rate of survival in TNBC patients (141). An investigation on the SDF-1/CXCR4 axis in a breast carcinoma model revealed their role of hypoxia. It was confirmed that in a hypoxic tumor niche activation of HIF leads to the transcription of an array of HIF target genes including SDF-1 and CXCR4 which contributes to tumor cell migration and adhesion to endothelial cells in breast cancer cells. Moreover, the study also illustrated that SDF-1 binding to CXCR4 stimulated tube formation in endothelial cells, which point towards its role in angiogenesis and trans-endothelial migration (142). Since metastasis and related poor survival is a hallmark of breast cancer, pathways targeting trans-endothelial migration could have a significant impact in clinical trials.

## 7 Recent strategies to overcome hypoxic endothelium driven immunosuppression

The hypoxic microenvironment and impaired immune response to cancer cells by innate immune cells continue to be major obstacles for immunotherapy. Recent advancements in nano-immunotherapy, however, might lessen immunosuppression brought on by hypoxia and enhance systemic antitumor immune responses to eradicate metastatic breast cancer cells. Similar to this, *in situ* O_2_ generation, O_2_ delivery, normalisation of tumor vasculature, and mitochondrial-respiration inhibition could be the alluring therapy to overcome hypoxia-driven immune suppression for preventing the growth and progression of metastatic breast cancer cell lines. Monoclonal antibodies, immune modulators, and biodegradable bio-nanomaterials could address the problems in a very satisfactory way when used in conjunction with such methodologies. Nowadays, there is a great deal of interest in using nanomaterials as treatments to target the downstream pathways that lead to hypoxia-driven immune suppression in breast cancer (143). In this sense, PD1 receptor antagonist delivered in nanoform has the potential to effectively address the problem of hypoxic endothelium-driven immunosuppression during the treatment of breast cancer. The specific ligand antagonist of the upstream hyper-expressive biomarkers, which is responsible for the immunosuppressive state caused by hypoxic endothelium around the breast cancer microenvironment, can be used to develop new strategies. This may be a new strategy to improve immune monitoring in the treatment of breast cancer, especially for managing disease progression. Additionally, by preventing hypoxia in TME, such an approach could activate the immune cells, strengthen immune surveillance, and destroy breast cancer cells. By disrupting the hypoxia-mediated cancer signaling pathway, they may specifically target the hypoxic TME (144). Through the generation of oxygen-derived free radicals, radiation destroys tumor cells by causing DNA fragmentation. Due to the absence of oxygen and a reduction in DNA repair processes, hypoxia confers resistance to radiation therapy and reduces the effectiveness of radiation. The main cause of chemotherapy resistance in hypoxic tumors is the fact that many of the commonly used medications need oxygen to release the deadly free radicals that kills the tumor cells (145). In addition, another study that looked at the connection between HIF-1α/CAIX and the response to epirubicin found a strong correlation between high HIF-1α expression and a subpar response to treatment (146). This gives a clear insight that hypoxic pathway is involved in significant events that have a direct impact on the efficacy of numerous treatment methods. In the earlier sections of the review, we addressed the role of pro-angiogenic factor stimulation that causes tumor-associated endothelial cells to become anergic, losing the capacity to react to inflammatory signals and rendering them unable to activate EAMs (79, 147). It was discovered that this anti-infiltration barrier helped tumors avoid being destroyed by the immune system. Consequently, it is thought that using angiogenesis inhibitors to encourage leukocyte infiltration into the tumor is a useful way to increase the effectiveness of ICIs (148, 149). Bevacizumab, a VEGFA neutralizing antibody, has been shown to increase the number and activation of DC (149–151) as well as the number of cytotoxic T cells (152, 153) and to reverse VEGF-induced T cell exhaustion (154). Whereas, Sutinib, a TKI of VEGFR and other kinases, was shown to decrease the number of MDSCs and T reg cell (155, 156). Xiaodong et al. (2020). identified a tumor endothelial specific marker CLEC14A, which specifically recruits T reg recruitment and subsequently enhance immune suppression in the TME. CLEC 14 A specific CART cells, exhibited substantial decrease in tumor growth through IFN-γ indicating their antitumor potential (157).

## 8 Future perspectives

TNBCs exhibit a markedly increased HIF transcriptional activity and a subpar response to the existing therapeutic strategies (158). Therefore, it makes sense to speculate that a novel therapeutic approach to treat TNBCs might involve targeting hypoxia in the TME. Preclinical investigations indicate that the combination of cytotoxic chemotherapy with molecules that block HIFs is particularly promising. Therapeutic studies with Digoxin and acriflavine, two HIF-1α inhibitors, demonstrated reduced initial tumor development, vascularization, invasion, and metastasis in breast cancer animal models. Furthermore, digoxin prevents HIF–1α dependent transcriptional responses that encourage cancer stem cell (CSC) resistance to chemotherapy, which causes tumor regression in TNBC when combined with paclitaxel or gemcitabine (159). Numerous synthetic and natural substances have been shown to block the regulation of HIF-1 on downstream target genes by lowering HIF-1 mRNA levels, accelerating the protein’s breakdown, and preventing HIF-1 and HIF-1 dimerization (160, 161) **(** **Table 2** **)**. An effective anticancer therapy may include normalizing the tumor vasculature rather than destroying it. Anti-angiogenic drugs must be dosed carefully during vascular normalization in order to reverse the aberrant phenotype of the tumor vasculature and increase blood flow and oxygenation. Through vessel maturation and the alleviation of immunosuppression brought on by hypoxia and/or VEGF, it has been demonstrated that vascular normalization enhances immunological responses (16, 162). It indicates that restoring the structural and functional integrity of the tumour vasculature is a viable method for polarising TAMs to an anti-tumor phenotype (163). The hypoxic tumor endothelium is hyperglycolytic, thus, targeting tumor endothelial metabolism might offer a novel therapeutic strategy. Glycolysis is the energy source for endothelial sprouting in angiogenesis rather than oxidative phosphorylation for ATP production. Thus, blocking PFKFB3, a key molecule involved in endothelial glycolysis pathway, reduced vessel sprouting and angiogenesis (164). VEGF and PFKFB3, which are both implicated in the TEC’s glycolytic pathway, were downregulated when tumor-cell-specific cyclooxygenase (COX-2) was pharmacologically inhibited. The restoration of glucose metabolism in TECs and the reduction of tip cells, filopodia, and branching are affected by COX-2 inhibitor therapy-induced inhibition of PFKFB3-mediated endothelial cell motility (165). The role of anti-angiogenic drugs in regulating the tumor vasculature and reducing hypoxia-induced immune suppression has been shown in a number of preclinical investigations. These anti-angiogenic therapies have also been successful in overcoming endothelial anergy, which results in normalized production of endothelial adhesion molecules, which are necessary for leukocyte trans-endothelial migration into the TMC. Treatment that targets the VEGF pathway may result in increased immune infiltration and ICAM1 overexpression in renal cell carcinoma (166). Since TECs have higher glycolytic rates than typical proliferating ECs, reducing glycolysis by blocking PFKFB3 with 3-(3-pyridinyl)-1-(4-pyridinyl)-2-propen-1-one (3PO) may be able to suppress tumor growth. According to studies, 3PO therapy increased the effectiveness of chemotherapy, aided in the normalisation of tumor vessels, tightened the EC barrier to prevent cancer metastasis, and encouraged tumor vessel normalisation. In mouse tumor models, proliferating ECs were treated with the weak mitochondrial uncoupler Embelin, which resulted in reduced mitochondrial oxidative phosphorylation, inhibited tumor growth, and reduced microvessel density (164). The endothelium of tumors significantly differs from endothelium of healthy tissues in a number of aspects, including metabolic reprogramming, alternative morphology, cytogenetics, and molecular genetics (82). So, a viable strategy to restore normal tumor vasculature involves attacking tumor endothelium. The research carried out by our group has also shown a rise in the infiltration of effector T cells, which in mouse TNBC inhibit the pro-angiogenic CXCR2 receptor. Targeting CXCR2 or other pro-angiogenic expressed on tumor endothelium may produce breakthrough outcomes in accordance with the promising results. (Unpublished data). Similar results were seen *in vitro* when a novel synthetic quinoline derivative was administered to block the pro-angiogenic chemokine (Unpublished data). Targeting the molecules involved in the regulation of tumors would open up the potential of a wide range of therapeutic options, normalising vascular function, and improving immune surveillance. Future therapeutic implications may benefit from knowledge of the variations in chemokines or pro-angiogenic substances produced by NECs and TECs in a hypoxic environment, as well as the expression of these receptors in normal and malignant cells.

**Table 2:** Hypoxia and vascular targeting compounds.

HIF Targeting Novel Compounds	Action	Vascular targeting Compounds	Action
Digoxin Acriflavine	Prevents HIF -1 α dependent transcriptional responses -Tumor regression Prevents dimerization of HIF-1α – Prevents tumor vascularization	Bevacizumab - VEGFA neutralizing antibody	Increase the number of DCs, Tc cells
Sanguinarine, Elemene (C15H24), Isoliquiritigenin (ISL), Cardamonin, Anhydroicaritin (AHI), Melittin (MEL), Fucoidan, Curcumin, Arsenic sulfide (As4S4), Acriflavine, Ganetespib, and Echinomycin	Prevents the dimerization of HIF-1α and HIF-1β	Sutinib - TKI of VEGFR	Decrease the number of MDSCs and Treg cells
		TEC specific marker - CLEC 14 A specific CART cells	Reduction in tumor growth via IFN-γ
		Tumor-cell-specific cyclooxygenase (COX)-2 Inhibitor	Downregulation of PFKFB3, involved in hyper-glycolysis of TEC Downregulation of VEGF
		PFKFB3 blocker - 3-(3-pyridinyl)-1-(4-pyridinyl)-2-propen-1-one (**3PO**)	Reducing hyper-glycolysis in TECs Normalisation of tumor vessels

## 9 Conclusion

In the light of the foregoing discussion, it will be wise to more effectively employ therapeutic alternatives in BCs with treatment resistance. Understanding the control of selective immune cell trafficking *via* hypoxic tumour endothelium may also be necessary. Clinical studies have found that the early preventative methods for the development of new blood vessels have only limited effects. The establishment of resistant mechanisms and enhanced tumor hypoxia are the causes of this insufficient efficacy. The development of effective treatment combinations can be facilitated by an understanding of how tumors use hypoxic endothelium cells to evade the immune system. Hypoxia regulating molecules and Immune Checkpoint Inhibitors (ICIs) should be combined to improve the prognosis of patients with hypoxic breast cancer in the light of the role played by the hypoxic TME in immune evasion. Therefore, molecules linked to selective trafficking may offer brand-new prognostic criteria to justify the use of particular immunotherapy regimens in conjunction with vascular targeting therapeutics. Though it will take time to investigate and comprehend such issues, promising research on anti-angiogenic adjuvant immunotherapy techniques offers hope for the improvement of treatment ways to improve the outcomes of breast cancer patients. A concerted effort from all investigators is imperative to usher in an era free from the woes of life-threatening diseases such as the BCs.

## Author contributions

SB and PP conceived the concept and contributed writing and editing of the review. All authors contributed to the article and approved the submitted version.

## Glossary

**Table:**

TME	Tumor Microenvironment
BC	Breast Cancer
HIF	Hypoxia Inducible Factor
VHL	Von-Hippel Lindau
EMT	Epithelial to mesenchymal transition
EMT-TFs	EMT transcription factors EMT transcription factors
TNBC	Triple negative breast cancer
TGF-β1	Transforming growth factor-β1
SMAD3	Suppressor of Mothers against Decapentaplegic
GLUTs	Glucose Transport Proteins
HK1, 2	Hexokinase 1 and 2
PDK1	Pyruvate Dehydrogenase Kinase 1
ECM	Extra Cellular Matrix
ROS	Reactive Oxygen Species
BCSCs	Breast cancer stem cells
MMP-2 and MMP-9	Metalloprotease 2 and 9
VEGF	Vascular Endothelial Growth Factor
TILs	Tumor Infiltrating Lymphocytes
TCR	T-cell receptor
IFN	Interferon
MHC	Major Histocompatibility Complex
MDSCs	Myeloid-Derived Suppressor Cells
Tregs	T regulatory cells
TH1	T-helper type 1
TH2	T-helper type 2
TAMs	Tumor-Associated Macrophages
NK cells	Natural Killer Cells
PDL-1	Programmed Death Ligand-1
CXCL12	C-X-C Motif Chemokine Ligand 12
CXCR4	C-X-C Motif Chemokine Receptor 4
CXCR12	C-X-C Motif Chemokine Receptor 12
CCL5	CC-chemokine Ligand 5
CCL2	CC-chemokine Ligand 2
bFGF	basic Fibroblast Growth Factor
PGF	Placental Growth Factor
Ang-2	Angiopoietin-2
ECs	Endothelial Cells
ICAM1	Intercellular Adhesion Molecule 1
VCAM1	Vascular Cell Adhesion Molecule 1
TNFα	Tumor Necrosis Factor α
IFNγ	Interferon γ
IL-1	Interleukin 1
PFKFB	6-phosphofructo-2-kinase/fructose-2,6-biphosphatase 3
Sca-1	stem cell antigen-1
MDR-1	Multi-Drug Resistance Mutation 1
ALDH	Aldehyde Dehydrogenase
NO	Nitric Oxide
GM-CSF	Granulocyte-Macrophage Colony-Stimulating Factor
PGE2	Prostaglandin 2
LECs	Lymphatic Endothelial Cells
SAA1	Serum Amyloid A1
TEM1	Tumor Endothelial Marker 1
CTLA4	Cytotoxic T Lymphocyte-associated Antigen 4
TCR	T cell receptor
HEVs	High Endothelial Venules
EndMT	Endothelial-mesenchymal transition
ICIs	Immune Checkpoint Inhibitors
